# First record of the Peters’s Pipistrelle *Hypsugo petersi* (Chiroptera, Vespertilionidae) from China and an updated identification key to Chinese *Hypsugo*

**DOI:** 10.3897/zookeys.1290.186733

**Published:** 2026-08-25

**Authors:** Yang Guo, Kun-hao Chen, Xin-dan Fan, Lin-ying Xue, Hu-hua Yang, Yuan-jie Xiao, Xiao-yun Wang, Yi Wu, Yang Yue, Wen-hua Yu

**Affiliations:** 1 South China Biodiversity Research Center, School of Life Sciences, Guangzhou University, Guangzhou, 510006, China South China Biodiversity Research Center, School of Life Sciences, Guangzhou University Guangzhou China; 2 Jinguang Temple Provincial Nature Reserve Administration Bureau, Yongping, Dali, 672600, China Jinguang Temple Provincial Nature Reserve Administration Bureau Dali China

**Keywords:** Chiropteran fauna, *Cytb*, distribution, morphology, morphometrics, *
Pipistrellus
*, Vespertilioninae

## Abstract

In July 2025, one small vespertilionid bat was collected from a building of Jinguang Temple Provincial Nature Reserve, Dali, Yunnan, China. Based on integrative analyses of morphological and morphometric characters, together with phylogenetic evidence based on the mitochondrial cytochrome *b* (*Cytb*) gene, we identified the specimen as *Hypsugo petersi*. Previously, this species has been documented only in Borneo and the Philippines; therefore its discovery represents the first record of *H. petersi* in China and the Asian mainland. Furthermore, an identification key to all Chinese *Hypsugo* species is presented to facilitate accurate species identification and to support future biodiversity surveys and conservation management.

## Introduction

The genus *Hypsugo* (Kolenati 1856) (Vespertilionidae, Chiroptera) comprises 22 species distributed across the southern Palaearctic, most of Africa and the Indomalayan region and south to Lesser Sunda lslands (Wilson and Mittermeier 2019). In China, six *Hypsugo* species have been recorded (Wei et al. 2025). The taxonomic status of *Hypsugo*, however, has long been controversial. Ognev (1928) historically included it within *Eptesicus* (Rafinesque, 1820), while Hill and Harrison (1987) classified *Hypsugo* as a subgenus of *Pipistrellus* (Kaup, 1829) based on baculum morphology. Nearly simultaneously, Horáček and Hanák (1986) listed several additional penile and dental traits supporting its recognition as a full genus. Subsequent authorities remained divided, with some treating it as a subgenus of *Pipistrellus* (e.g., Tate 1942; Corbet and Hill 1992; Koopman 1994) and others, drawing on karyological, isozyme, and genetic data, advocating for its generic distinction (e.g., Ruedi and Arlettaz 1991; Volleth and Heller 1994; Roehrs et al. 2010). Simmons (2005) integrated karyotype and available molecular data at the time to propose the *Pipistrellus* and *Hypsugo* belong to two different tribes within Vespertilioninae (Vespertilionini and Pipistrellini, respectively). This generic rank is now widely accepted (e.g., Francis 2008; Goodman et al. 2015).

In spite of the recently published work of Wei et al. (2025), it is important to note that our knowledge of the larger-bodied species of *Hypsugo* in China (formerly placed in *Falsistrellus* (Troughton, 1943), e.g., *affinis*, *mordax*, and *petersi*) remains limited. In particular, no rigorous published study has yet unequivocally confirmed the presence of either *H. mordax* or *H. affinis* in China.

Peters’s Pipistrelle bat, *H. petersi*, was described as *Vespertilio petersi* (A.B.Meyer, 1899) from specimens collected in Minahassa, northern Sulawesi, Indonesia. Tate (1942) assigned it to the “*affinis*-group” within *Pipistrellus*, which also included two other species: *P. affinis* (Dobson, 1871) and *P. mordax* (Peters, 1866). Troughton (1943) later erected the genus *Falsistrellus* based on Australian species. Then Hill and Harrison (1987) placed *Falsistrellus* in the subgenus *Pipistrellus* and transferred the “*affinis*-group” to this subgenus. Concurrently, they noted that “…*Pipistrellus* (*Falsistrellus*) appears to be related to *P.* (*Hypsugo*) of which it may be the eastern representative…” (Hill and Harrison 1987). Simmons (2005) elevated Asian and Australian populations of *Falsistrellus* to generic rank and transferred *petersi* accordingly. Based on a comprehensive study by Görföl and Csorba (2018) integrating molecular (*CO1*, *Cytb* and *Rag2* segments), cranial, dental, and multivariate statistical evidence, the Asian members of *Falsistrellus*, including *petersi*, were formally transferred to the genus *Hypsugo*, a taxonomic revision adopted by Wilson and Mittermeier (2019).

Despite its early description (Meyer 1899), the species is known from very few localities, with confirmed records limited to Borneo and the Philippines, resulting in a paucity of specimens (Wilson and Mittermeier 2019). Consequently, it was classified as Data Deficient on the International Union for Conservation of Nature (IUCN) Red List of Threatened Species (assessed as *Falsistrellus petersi*), reflecting the paucity of records across its purported extensive geographic range (https://www.iucnredlist.org/species/17359/22125873).

During a series of chiropteran surveys in southwestern Yunnan Province, China, in July 2025, a single vespertilionid individual was captured via sweep netting. Based on morphology, morphometric analyses, and phylogenetic inference using *Cytb* sequences, it was identified as *H. petersi*. This finding represents the first record of this rare species in China and constitutes a new species record for the genus *Hypsugo* in China. Notably, it also represents the seventh *Hypsugo* species documented in China. In this study, we provide details of this discovery, present new distribution information and provide an up-to-date key to identify all *Hypsugo* species occurring in China.

## Material and methods

### Sample collection

**Figure 1.**
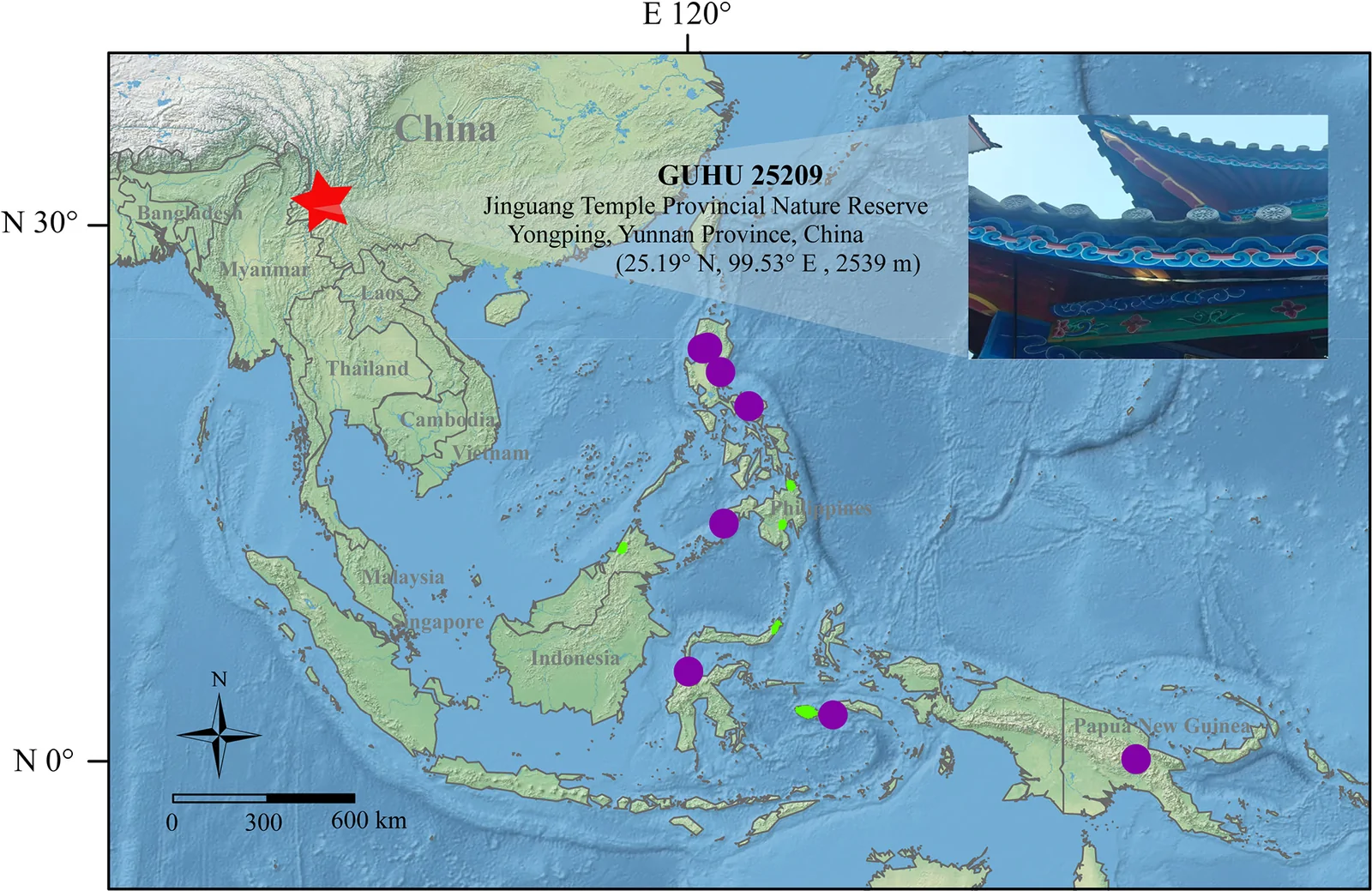
Global distribution records of *Hypsugo petersi* (distribution data from GBIF, IUCN and this study). Purple dots indicate historical records; red asterisks mark the locations surveyed in this study; green shading indicates the known distribution area of *H. petersi*.

In July 2025, a vespertilionid bat (♀, GZHU 25209) was collected using sweep nets from the eaves of the management station building within the Jinguang Temple Provincial Nature Reserve in Dali, Yunnan Province, China (25°11'55"N, 99°31'50"E (DMS), ca 2500 m) (Fig. 1). All field survey and sample collection protocols complied with the current laws of China. Handling of live animals followed American Society of Mammalogists guidelines (Sikes 2016). The voucher specimen was determined to be an adult based on the degree of epiphyseal-diaphyseal fusion (Brunet-Rossinni and Wilkinson 2009). The specimen was preserved in 75% ethanol and deposited at the South China Biodiversity Research Center, School of Life Sciences, Guangzhou University, Guangdong Province, China.

### Morphological measurements and morphometric analysis

External and craniodental measurements were taken with digital calipers accurate to 0.01 mm (MNT-150, Shanghai Menet Industrial Co., Ltd). External measurements include: body length (**HB**); tail length (**T**); ear length (**E**); hind foot length (without claw) (**HF**); forearm length (**FA**); tibia length (**Tib**). Skull measurements include: greatest length of skull (**GTL**); condylocanine length (**CCL**); condylobasal length (**CBL**); braincase width (**BCW**); braincase height (**BCH**); zygomatic width (**ZYW**); interorbital width (**IOW**); maxillary toothrow length (**C^1^M^3^L**); width across upper canines (**C^1^C^1^W**); width across upper molars (**M^3^M^3^W**); mandibular toothrow length (**LC_1_M_3_L**); greatest length of mandible (**ML**). These external and craniodental measurements followed those from Yu et al. (2020) for consistency.

The right wing was pinned flat against a foam board and photographed perpendicular to the plane. The image was saved in jpg format, and wing morphometric variables (total wing area, wingspan, palmar-membrane area and brachial-membrane area) were measured with BAT-WINGAREA (Yue et al. 2019) with a precision of 0.1 mm^2^. Then, we calculated the three primary airfoil parameters: wing loading (**WL**), aspect ratio (**A**), wing tip-shape index (**I**). The calculated parameters were assigned to eco-morphological categories according to the criteria of Norberg and Rayner (1987) and Jennings et al. (2004).

Due to the lack of research on Chinese *Hypsugo*, we supplemented external morphometric data for four Chinese *Hypsugo* species (*H. alaschanicus* (Bobrinskii, 1926), *H. cadornae* (Thomas, 1916), *H. pulveratus* (Peters, 1871), and *H. affinis*) and cranial measurements for three of them (*H. alaschanicus*, *H. cadornae*, *H. pulveratus*). Data were compiled from our specimens and literature (sample number see Table 1). Based on these morphometric data, we performed principal component analysis (PCA) using the “psych” package in R (Jombart 2008). Due to a lack of morphological and cranial data for *H. mordax* and taxonomic uncertainty for *H. savii*, both species were excluded from the analysis.

**Table 1.** Species information for PCA analysis. “—” indicates a missing value.

**Species**	**External**	**Craniodental**
*Hypsugo petersi*	GZHU 25209	GZHU 25209
*Hypsugo alaschanicus*	GZHU 25694	GZHU 25694
	GZHU 25695	GZHU 25695
*Hypsugo cadornae*	GZHU 22439	GZHU 22439
	GZHU 22440	GZHU 22440
	GZHU 23869	GZHU 23869
	GZHUhn 24018	GZHUhn 24018
	GZHU 24018	GZHU 24018
	GZHU 24496	GZHU 24496
*Hypsugo pulveratus*	GZHU 15092	GZHU 15092
*Hypsugo affinis*	G. Edirisinghe et al. (2020) (*N* = 3)	—

### Phylogenetic analysis

Total DNA of each sample was extracted using the DNU333-03 Animal Genome Extraction Kit (Maibao Biotechnology, China). The mitochondrial cytochrome *b* (*Cytb*) gene of 1140 bp was amplified and sequenced with *Cytb*-F (5’-TAG AATATCAGCTTTGGGTG-3’) and *Cytb*-R (5’-AAATCACCGTTGTACTTCAAC-3’) primers. We aligned our sequence with publicly available data from other species of *Hypsugo*, *Laephotis*, *Nycticeinops*, *Tylonycteris*, *Vespertilio*, *Nyctalus*, *Scotoecus*, *Pipistrellus* and *Falsistrellus* using MUSCLE v. 5.3 (Edgar 2022). *Myotis chinensis* was designated as the outgroup. Maximum likelihood (ML) inference in IQ-TREE v. 2 (Nguyen et al. 2015) was used for phylogenetic reconstruction, with a TIM2+F+I model as best substitution model. Branch support was estimated with 1000 replicates using the ultrafast bootstrap algorithm (UFBoot) implemented in IQ-TREE. Pairwise K2P distances for the *Hypsugo* dataset were estimated in MEGA v. 11 (Tamura et al. 2021) with 1000 bootstrap replicates to estimate standard errors.

## Results

### Morphological characteristics

**Figure 2.**
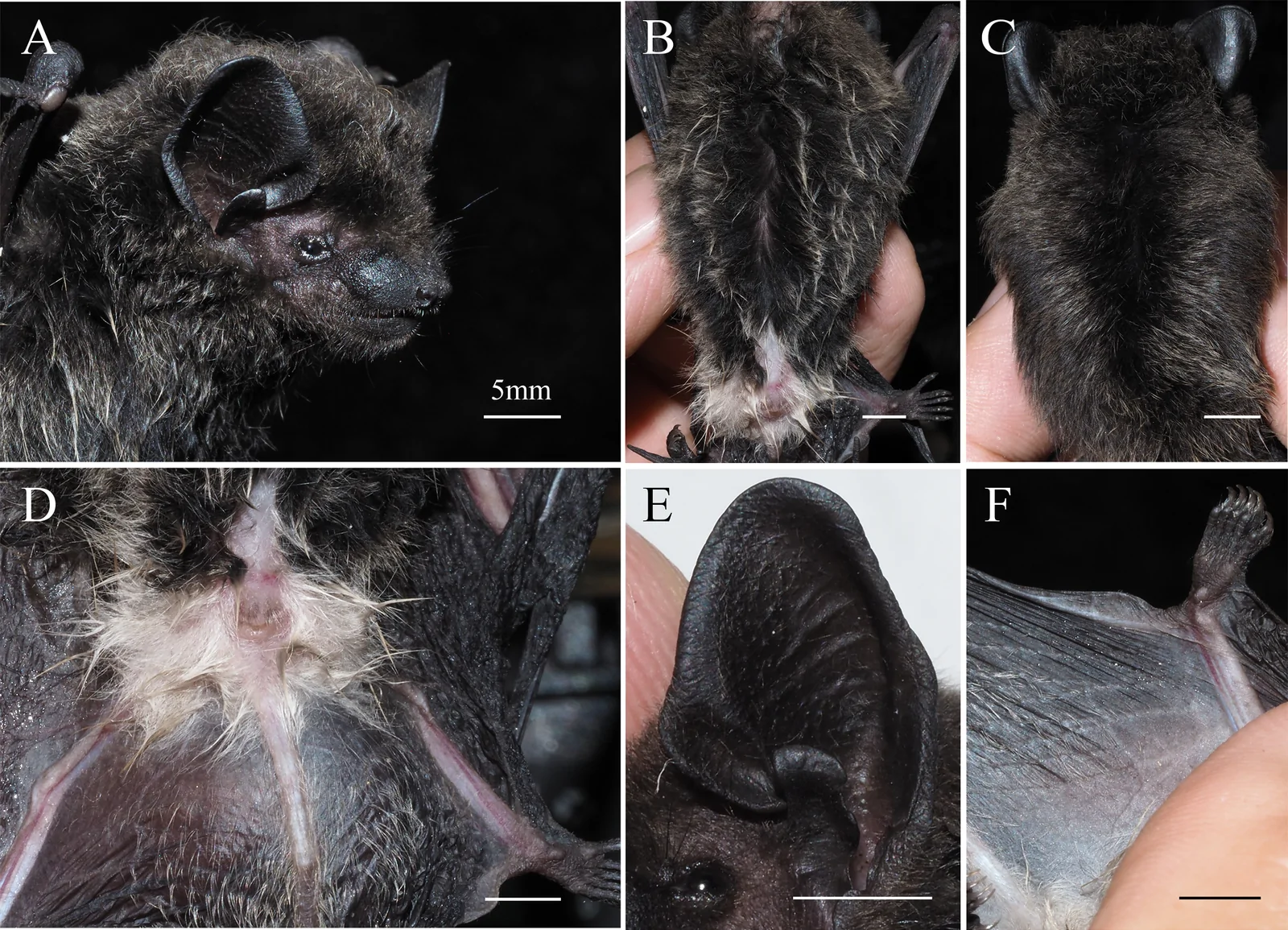
Photographs of *Hypsugo petersi* (♀, GZHU 25209) from Yunnan, China. **A**. Lateral view; **B**. Ventral pelage; **C**. Dorsal pelage; **D**. Hair on the femoral surface and perianal region; **E**. Tragus; **F**. Hair on the plagiopatagium, calcar lobe. Scale bar: 5 mm (in **A**); (**B–F**) share the same scale.

It is a large-sized species in *Hypsugo* with an FA of 39.94 mm. Dorsal fur is long and woolly, upper parts are dark chocolate-brown with lighter tips, giving a frosted appearance; ventral fur is paler with brown tips (Fig. 2B, C). Wing membranes are blackish and hairless, except for a small patch of pale-brown fur that extends onto the ventral surface of the femur and the area adjacent to the anus (Fig. 2D). The ears are dark, broad, and rounded. Tragus is short, broad and blunt, usually slightly curved anteriorly (Fig. 2E). The muzzle is dark brown, with simple, non-tubular nostrils (Fig. 2A). The tail tip extends beyond the tail membrane. The calcar bears a slight keel extending medially from the hindfoot (Fig. 2F). Following Norberg and Rayner (1987) and Jennings et al. (2004), the wing of *H. petersi* is characterized by high wing loading (~16.77 m^2^), average aspect ratio (~6.87) and a low tip-shape index (~0.77).

**Figure 3.**
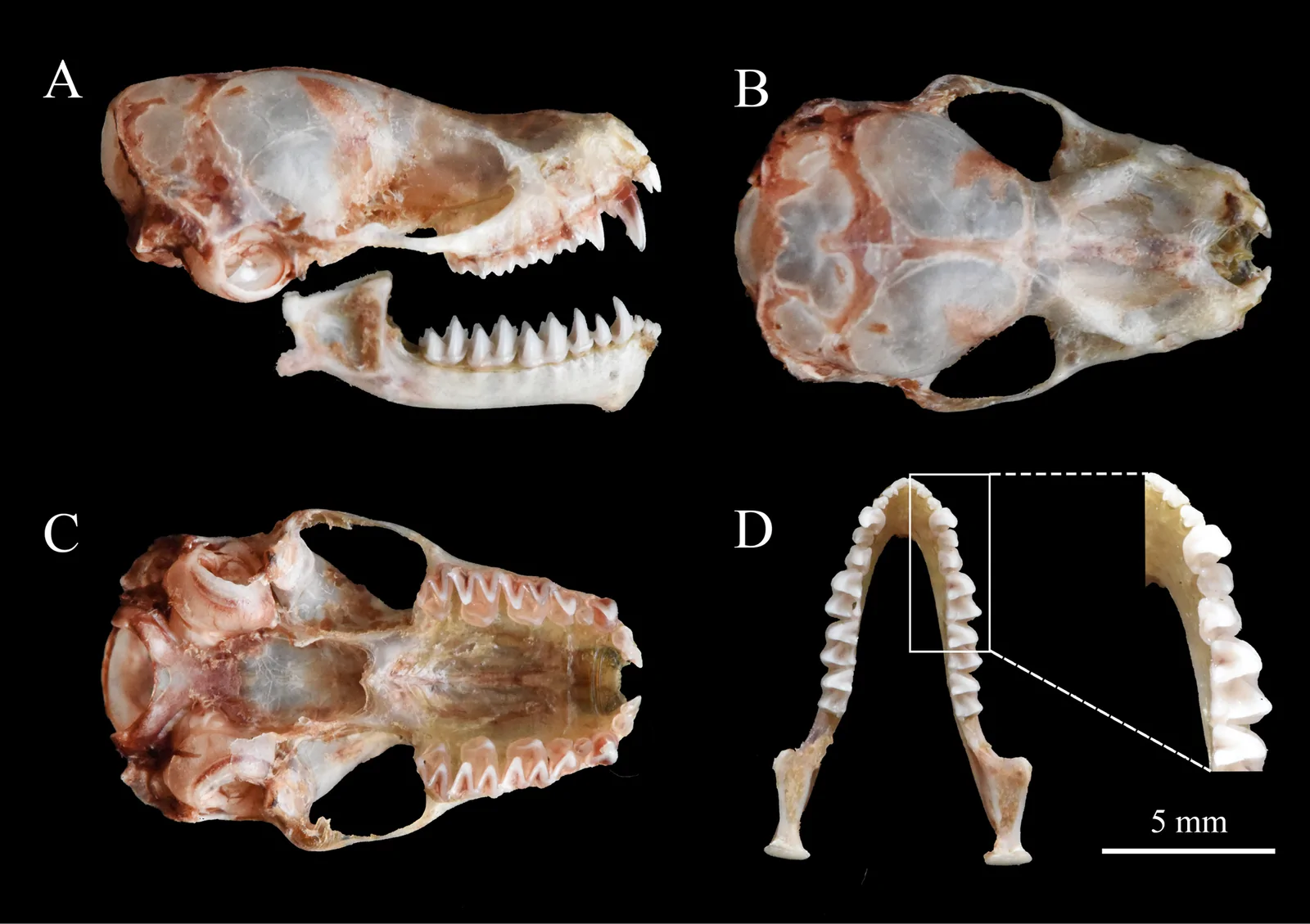
Craniodental morphology of *Hypsugo petersi* (♀, GZHU 25209) from Yunnan, China. **A**. Lateral view of skull and mandible; **B**. Dorsal view of skull; **C**. Ventral view of skull; **D**. Frontal view of mandible.

Skull is long and narrow, with slender zygomatic arches (Fig. 3B). Occipital crests are well developed, sagittal and lambdoidal crests are present (Fig. 3B). Upper premolars are intruded and partially reduced. Outer upper incisor is not greatly reduced, only slightly smaller than the inner one, and possesses minute supplementary cusps. The upper canine is without supplementary cusps, while lower molars are of the myotodont type, with talonid exceeding trigonid in size; the posterior ridge connects the lower buccal cusp and the lower lingual cusp with the lesser buccal cusp separated (Figs 3C, 6A). Condylocanine length (**CCL**) is 14.42 mm, and maxillary tooth (**ML**) row length is 5.75 mm (Table 2).

**Table 2.** External and craniodental measurements (mm) and body mass (g) of *Hypsugo petersi*, and variable loadings on principal components (PCs) (unit: g, mm). Independent principal component analyses (PCAs) were performed separately on the external and craniodental measurements data, and the resulting loadings were presented in the same column. “—” indicates missing values.

**Characters**	**China (this study)** ♀, ***N* = 1**	**Indonesia, Malaysia, Philippines (Görföl and Csorba 2018) *N* = 5–10**	**Philippines (Heaney et al. 2012) *N* = 3**	**PC1s**	**PC2s**
MASS	7.5	—	6.0–6.6	—	—
HB	50.39	—	—	0.85	0.25
T	41.57	—	35–39(3)	0.26	0.83
E	15.19	—	14–15(3)	0.92	0.04
HF	8.49	—	9	0.45	0.22
FA	39.94	38.5–41.9 (5)	38–39	0.51	0.59
Tib	14.90	—	—	0.07	0.93
CBL	14.86	—	—	0.88	0.36
ML	11.49	10.51–11.48 (9)	—	0.85	0.30
LC_1_M_3_L	6.10	5.75–6.23 (10)	—	0.84	0.22
BCW	7.48	6.82–7.61 (9)	—	0.83	-0.07
CCL	14.88	13.56–14.67 (9)	13.48–13.81(3)	0.81	0.38
M^3^M^3^W	6.81	6.15–6.74 (9)	—	-0.73	-0.06
IOW	3.94	3.61–4.04 (10)	—	0.63	-0.51
C^1^M^3^L	5.68	5.37–5.82 (10)	5.35–5.42(3)	0.58	0.61
GTL	15.77	15.01–16.05 (5)	—	0.27	0.76
ZYW	9.66	9.11–9.97 (10)	9.09–9.21(3)	0.21	0.78
C^1^C^1^W	4.86	4.49–5.00 (10)	—	0.10	0.83
BCH	5.14	4.80–5.11 (5)	—	0.01	0.83

### Phylogenetic relationships and Genetic distance

**Figure 4.**
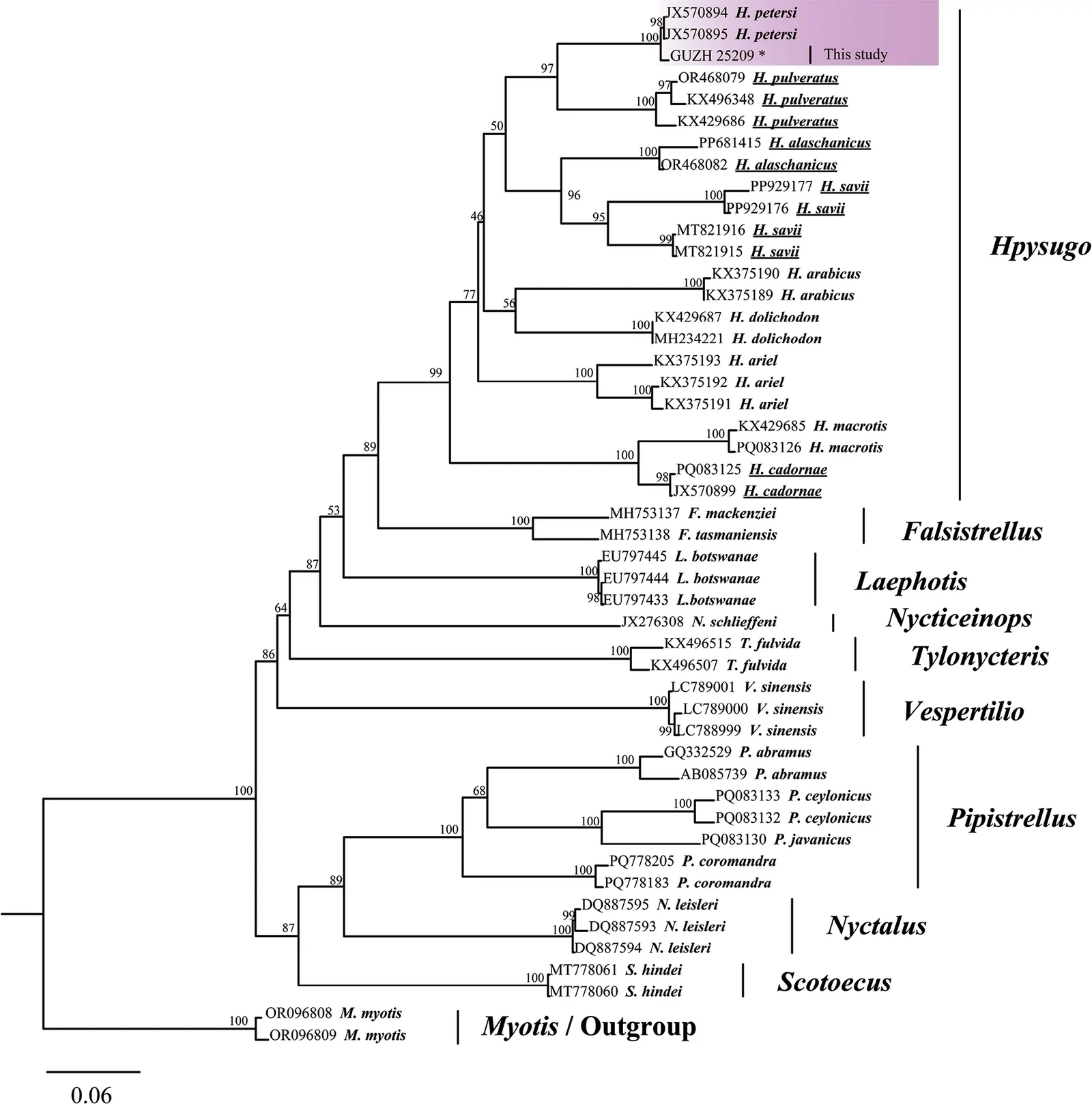
Phylogenetic relationships of close *Hypsugo* species based on *Cytb* sequences. The individual marked with an asterisk is the *H. petersi* investigated in this study; the purple rectangular box indicates *H. petersi*; the serial number before other species names is the *Cytb* accession number of the corresponding individual in National Center for Biotechnology Information (NCBI); the values on the branches represent the ultrafast bootstrap; the underlined *Hypsugo* species are those distributed in China.

The ML tree based on the *Cytb* gene strongly supports clustering this specimen with *H. petersi* (ultrafast bootstrap values = 100) (Fig. 4). *Hypsugo petersi* and *H. pulveratus* form a well-supported sister group (BS = 97). Using the K2P model, we calculated pairwise *Cytb* distances between the newly obtained *Hypsugo* sequences and all congeneric sequences available in GenBank. The distance of our sample from *H. petersi* on Luzon Island, Philippines was 0.10, whereas distances to other *Hypsugo* species ranged from 0.12 to 0.29 (Table 3).

**Table 3.** Genetic distance estimates between *Cytb* sequences.

**Species**	**1**	**2**	**3**	**4**	**5**	**6**	**7**	**8**	**9**	**10**
*Hypsugo dolichodon*										
*Hypsugo petersi*	0.12									
“*Falsistrellus petersi*” JX570894	0.12	0.10								
“*Falsistrellus petersi*” JX570893	0.12	0.10	0.00							
*Hypsugo alaschanicus*	0.15	0.14	0.13	0.13						
*Hypsugo savii*	0.15	0.14	0.15	0.14	0.14					
*Hypsugo ariel*	0.13	0.17	0.19	0.18	0.18	0.21				
*Hypsugo arabicus*	0.16	0.16	0.14	0.16	0.16	0.20	0.20			
*Hypsugo macrotis*	0.18	0.23	0.20	0.23	0.23	0.25	0.20	0.25		
*Hypsugo cadornae*	0.14	0.18	0.17	0.18	0.18	0.21	0.16	0.18	0.08	
*Hypsugo pulveratus*	0.17	0.17	0.16	0.16	0.16	0.21	0.19	0.19	0.23	0.18

### Multivariate comparison analysis

**Figure 5.**
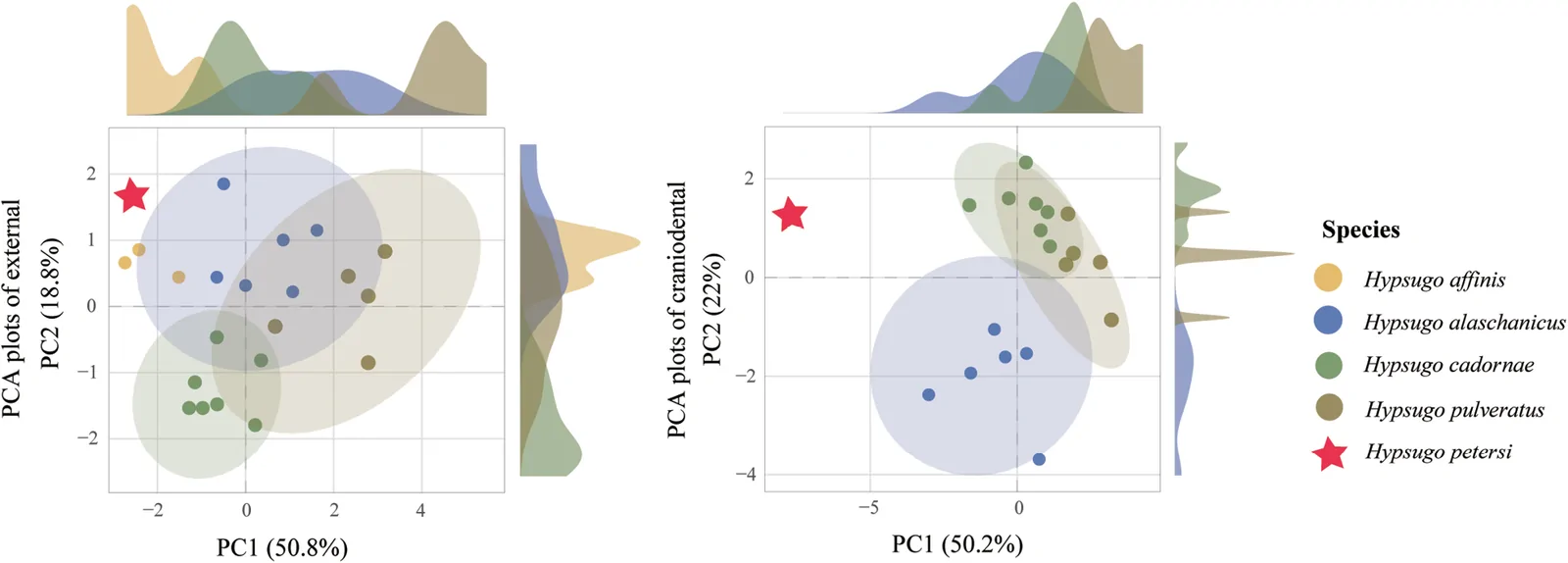
Two-dimensional PCA plots of *Hypsugo* species based on 12 craniodental and six external measurements. **A**. PCA plots of external for *H. alaschanicus*, *H. cadornae*, *H. pulveratus*, *H. affinis* and *H. petersi*, showing specimen projections and component loadings on the first two principal components; **B**. PCA plot of craniodental measurements for *H. alaschanicus*, *H. cadornae*, *H. pulveratus* and *H. petersi*, showing specimen projections and component loadings on the first two principal components.

PCA based on six external measurements revealed 69.6% of the total variance from the first two principal components (PCs) (35.5% and 34.1% for PC1 and PC2, respectively). Five morphological groups emerged in the PCA scatter plot (Fig. 5A). For PC1 and PC2 all measurements had positive loadings (Table 2). Ear length was characterized by higher PC1 scores; thus, specimens of *H. petersi* clustered to the right side compared with those of other taxa (Fig. 5A). Tibia length was characterized by higher PC2 scores; thus, specimens of *H. cadornae* clustered to the upward region compared with those of other taxa (Fig. 5A). PCA based on 12 craniodental measurements revealed 72.1% of the total variance from the first two principal components (PCs) (41.6% and 30.5% for PC1 and PC2, respectively). Four morphological groups were revealed in the scatter plot (Fig. 5B). For PC1, all measurements had positive loadings except M^3^M^3^W (Table 2). For PC2, all measurements had positive loadings except BCW, M^3^M^3^W and IOW (Table 2). Condylobasal length was characterized by higher PC1 scores; width across upper canines was characterized by higher PC2 scores; thus, specimens of *H. petersi* clustered to the upper right compared with those of other taxa (Fig. 5B).

## Discussion

### Morphological and molecular diagnosis of *H. petersi*

**Figure 6.**
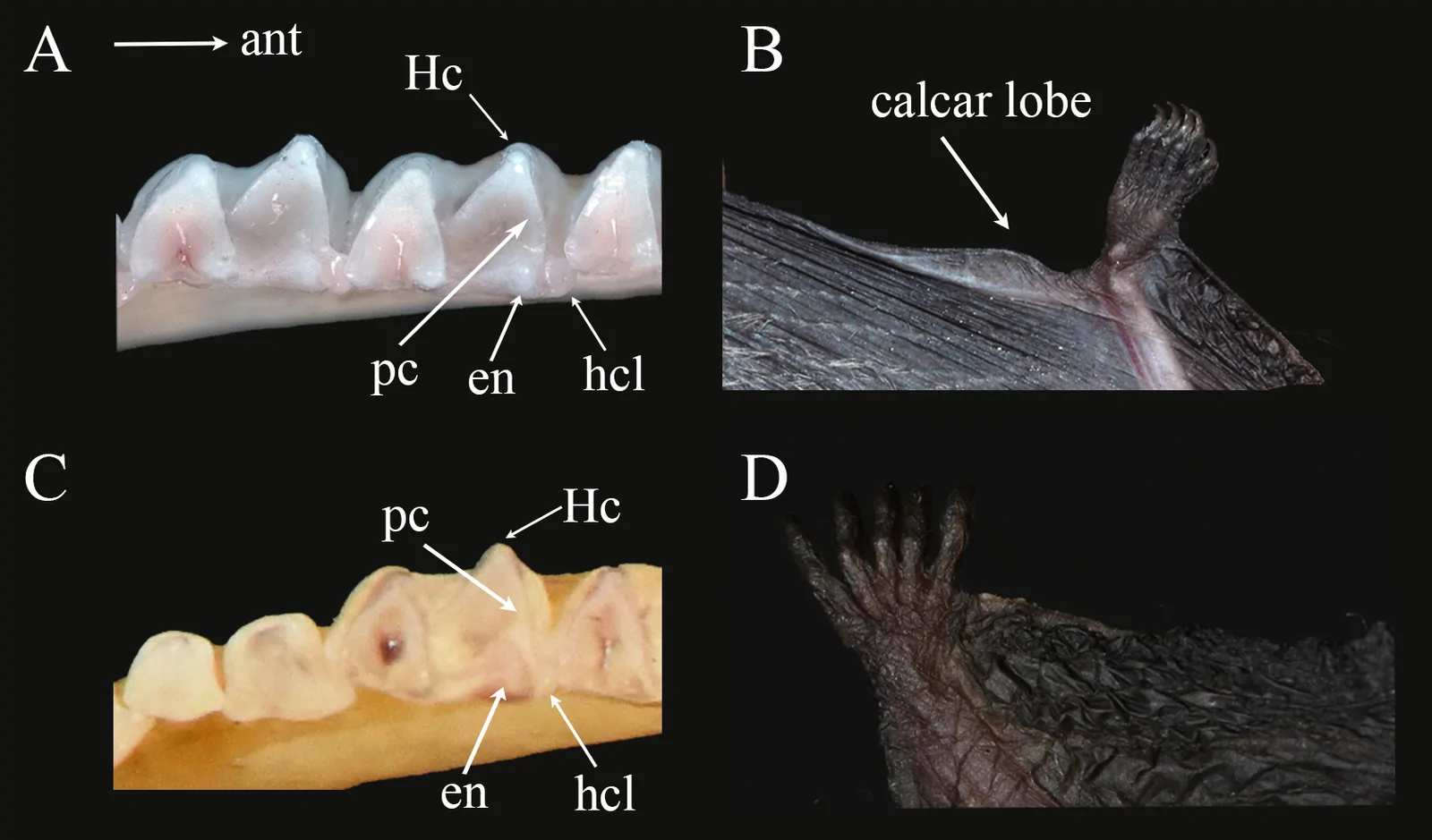
Comparison of lower molar and calcar characteristics between *Hypsugo* (**A, B**) and *Pipistrellus* (**C, D**). **A, C**. *H. petersi*; **B, D**. *P. abramus*. Hc: hypoconid, pc: postcristid, en: entoconid, hcl: hypoconulid; ant: anterior side.

Our specimen exhibited the key diagnostic characters of the genus *Hypsugo*: a calcar with a well-developed lobe, possessing a transverse cartilaginous septum (Figs 2F, 6B); lower molars are of the myotodont type, the talonid exceeds the trigonid in size (Figs 3D, 6A), which is consistent with the description by Kruskop (2013). Furthermore, it exhibits the typical characters of *H. petersi*, including: dorsal fur with paler tips, giving a frosted appearance; a small patch of pale-brown fur on the ventral surface of the femur and the perianal region, with a few hairs also sparsely scattered on the plagiopatagium adjacent to the calcar (Fig. 2F) (Wilson and Mittermeier 2019). Although some craniodental and external measurements were slightly larger than those previously reported (Table 2), the molecular phylogenetic tree based on the *Cytb* gene placed our sequence within a highly supported clade (BS = 100) alongside published “*F. petersi*” sequences (now as *H. petersi*; JX570895 and JX570894) (Heaney et al. 2012). Principal component analysis (PCA) based on craniodental and external measurements clearly separated this individual from all known *Hypsugo* species in China (Fig. 5). Therefore, based on morphological, molecular, and multivariate evidence, we identify this specimen as *H. petersi*.

### An updated key for Chinese *Hypsugo*

Owing to historically unstable taxonomic relationships among *Hypsugo*, *Pipistrellus*, and *Falsistrellus*, no comprehensive key for Chinese *Hypsugo* species has been established to date. “A Guide to the Mammals of China” (Smith and Xie 2008) includes a classification key for only three species: *H. pulveratus*, *H. alaschanicus* and *H. savii*. Following the transfer of Asian *Falsistrellus* species to *Hypsugo* by Görföl and Csorba (2018), the number of Chinese *Hypsugo* species increased by five. Subsequently, *H. cadornae* was reported from Guangdong, China (Xie et al. 2021), but no updated key was provided. Given the critical importance of identification keys for species delimitation and field surveys, we herein present a comprehensive key to all seven Chinese species of *Hypsugo* (see below in this section, provided in both English and Chinese). It should be noted that, owing to the currently limited knowledge of the large-bodied *Hypsugo* species (formerly assigned to *Falsistrellus*), and because no rigorous published study has yet confirmed the presence of *H. affinis* and *H. mordax* in China, these species are explicitly annotated in our identification key as requiring further verification.

### A key to the *Hypsugo* species occurring in China

(“cf.)

**Table:** 

1	Size larger, forearm length more than 38 mm	**2**
–	Size smaller, forearm length less than 38 mm	**4**
2	The fur on the ventral surface of the femur and around the anus is white brown, small amount of pale-brown hair is present near the plagiopatagium	***H. petersi***
–	The fur on the ventral surface of the femur and around the anus is not pale brown., pale-brown hair is absent around the plagiopatagium	**3**
3	Size smaller, forearm length 38–40 mm; canine directly contacts the third upper premolar (P^4^)	***H. affinis* (cf.)**
–	Size larger, forearm length 40–42; canine does not contact the third upper premolar (P^4^)	***H. mordax* (cf.)**
4	First upper premolar (P^2^) is displaced out the tooth row and markedly reduced in size and crown heigh	***H. cadornae***
–	First upper premolar (P^2^) is positioned superior to the tooth row and slightly medially depressed	**5**
5	First upper premolar (P^2^) is visible in lateral view; canine does not contact the third upper premolar (P^4^)	***H. pulveratus***
–	First upper premolar (P^2^) is not visible in lateral view; canine directly contacts the third upper premolar (P^4^)	**6**
6	Tragus widest near base, with one basal lobe	***H. alaschanicus***
–	Tragus widest above the middle, with two basal lobes	***H. savii***

### Wing morphology and habitat use

Wing morphology parameters (e.g., wing loading, aspect ratio, tip index) are key functional traits in bats, providing insight into flight performance and foraging ecology (Norberg and Rayner 1987; Jennings et al. 2004). Bats with high wing loading and aspect ratio exhibit faster flight speeds but reduced agility and maneuverability, favoring rapid flight in open habitats. In contrast, those with low wing loading and aspect ratio fly more slowly but with greater agility, facilitating navigation and foraging in structurally complex environments (Norberg and Rayner 1987; Jennings et al. 2004). Our result indicates that *H. petersi* exhibited high wing loading (~16.77 N m^2^), average aspect ratio (~6.87) and a low tip-shape index. This suite of traits suggests a flight style characterized by moderately high speed and limited maneuverability, suited for detecting and foraging along semi-open corridors such as forest edges and gaps. This inference is consistent with our field observation of the individual roosting in a building crevice surrounded by tall, dense coniferous forest at an elvation of 2539 m. It also aligns with prior habitat descriptions in “Handbook of the Mammals of the World – Bats” (Wilson and Mittermeier 2019): “…in the Philippines, specimens were caught in primary and heavily disturbed mossy forests and pine forests at high elevations (1,200–2,180 m)…” and “…In Borneo (Crocker Range) and the Philippines it was found roosting under the eaves of houses…”. Notably, our record extends the known altitudinal limit upward by 400 m, providing new evidence for its occupation of high-elevation niches.

### Distribution implication

Previously, *H. petersi* was known from scattered localities in the Philippines (Luzon and Mindanao), Borneo (Sabah and Sarawak), northern and central Sulawesi, and the Moluccas (Buru and Ambon Islands) (Wilson and Mittermeier 2019) (Fig. 1). This new record comes from southern Yunnan, China, which lies within a region adjacent to the Indo-China Peninsula biodiversity hotspot (including Laos, Myanmar, and Vietnam) (Zhang et al. 2023). The absence of records from the extensive Indo-Chinese Peninsula indicates a significant distributional gap and suggests that the species’ full geographic range remains poorly understood, necessitating targeted surveys and molecular screening.

In addition, our phylogenetic tree indicates that the Chinese sample forms the basal clade relative to other conspecifics (Fig. 4), potentially suggesting an older, possibly ancestral, lineage for this region. *Hypsugo petersi* is currently classified as Data Deficient on the IUCN Red List (Görföl et al. 2016), with critical gaps in knowledge regarding population trends, detailed ecology, and primary threats. Our discovery underscores the urgent need for dedicated monitoring and conservation planning for this enigmatic species.

In summary, this discovery represents a significant range extension for *H. petersi*, adds a new species to the Chinese chiropteran fauna, and highlights that bat diversity in China remains incompletely documented. To facilitate future research and conservation, we provide an updated identification key for Chinese *Hypsugo* species. We advocate for intensified integrative taxonomic studies and systematic field surveys to better elucidate the diversity and distribution of bats in this region.

## Additional information

### Artificial Intelligence (AI) use

The authors accept full responsibility for the content of the manuscript, including the disclosure of any use of AI.

No AI tools were used in the preparation of this manuscript.

### Author ORCIDs

K.-h. Chen https://orcid.org/0009-0000-2257-6666

L.-y. Xue https://orcid.org/0009-0002-6732-1901

### Data availability

All of the data that support the findings of this study are available in the main text or Supplementary Information.

## Supplementary materials

Supplementary material 1 A key to the *Hypsugo* species occurring in China (both in English and Chinese) Data type pdf Explanation note A key to the Hypsugo species occurring in China (both in English and Chinese) incliude *H. affinis*, *H. alaschanicus*, *H. cadornae*, *H. mordax*, *H. pulveratus*, *H. savii* and *H. petersi*. https://binary.pensoft.net/file/1753777This dataset is made available under the Open Database License (http://opendatacommons.org/licenses/odbl/1.0/). The Open Database License (ODbL) is a license agreement intended to allow users to freely share, modify, and use this Dataset while maintaining this same freedom for others, provided that the original source and author(s) are credited. Yang Guo, Kun-hao Chen, Xin-dan Fan, Lin-ying Xue, Hu-hua Yang, Yuan-jie Xiao, Xiao-yun Wang, Yi Wu, Yang Yue, Wen-hua Yu
