# Supplementary material for: First record of the Peters’s Pipistrelle Hypsugo petersi (Chiroptera, Vespertilionidae) from China and an updated identification key to Chinese Hypsugo
Source: Zookeys. 2026 Aug 25;1290:171–84. doi: 10.3897/zookeys.1290.186733 (PMC13535503; doi:10.3897/zookeys.1290.186733)
Supplement: Supplementary file 1 [file zookeys-1290-171_article-186733__-s001.pdf]

A key to the *Hypsugo* species occurring in China (in English) ("cf." indicates that the occurrence of the species in China requires further confirmation)

1. Size larger, forearm length more than 38 mm··**2**
   Size smaller, forearm length less than 38 mm··**4**
2. The fur on the ventral surface of the femur and around the anus is white brown, small amount of pale-brown hair is present near the plagiopatagium······························ ***H. petersi***
   The fur on the ventral surface of the femur and around the anus is not pale brown., pale-brown hair is absent around the plagiopatagium···**3**
3. Size smaller, forearm length 38 - 40 mm; canine directly contacts the third upper premolar (P^4)··***H. affinis*****（cf.）**
   Size larger, forearm length 40 – 42; canine does not contact the third upper premolar (P^4)··***H. mordax*****（cf.）**
4. First upper premolar (P^2) is displaced out the tooth row and markedly reduced in size and crown heigh··***H. cadornae***
   First upper premolar (P^2) is positioned superior to the tooth row and slightly medially depressed ··**5**
5. First upper premolar (P^2) is visible in lateral view; canine does not contact the third upper premolar (P^4)··***H. pulveratus***
   First upper premolar (P^2) is not visible in lateral view; canine directly contacts the third upper premolar (P^4)··**6**
6. Tragus widest near base, with one basal lobe···································***H. alaschanicus***
   Tragus widest above the middle, with two basal lobes·····························***H. savii***

中国高级伏翼属 *Hypsugo* 分种检索表（中文）(cf.”表示该物种在中国的分布还有待进一步确认)

1. 体型较大，前臂长 38 mm 以上……………………………………………………………**2**
   体型较小，前臂长 38 mm 以下……………………………………………………………**4**
2. 股骨腹侧表面及肛门附近毛发呈白棕色，距膜处有少量浅褐色毛发………………………………………………………………彼氏伏翼 ***H. petersi***
   股骨腹侧表面及肛门附近毛发非浅棕色，腹侧与距膜处无毛发……………………**3**
3. 体型较小，前臂长 38-40 mm；犬齿与第二上前臼齿（P^4）接触…………………………………………………………茶褐伏翼 ***H. affinis*（cf.）**
   体型较大，前臂长 40-42 mm；犬齿与第二上前臼齿（P^4）不接触…………………………………………………………大灰伏翼 ***H. mordax*（cf.）**
4. 第一上前臼齿（P^2）位于齿列外侧，相对较小且短………………卡氏伏翼 ***H. cadornae***
   第一上前臼齿（P^2）位于齿列上或略为内侵……………………………………………**5**
5. P^2 从侧面可见，犬齿与 P^4 不接触……………………………………灰伏翼 ***H. pulveratus***
   P^2 从侧面不可见，犬齿与 P^4 接触……………………………………………………**6**
6. 耳屏近基部最宽，下方有 1 基叶……………………………阿拉善伏翼 ***H. alaschanicus***
   耳屏中部以上最宽，下方有 2 基叶……………………………………萨氏伏翼 ***H. savii***
